# *In Silico* and *In Vitro* studies of taiwan chingguan yihau (NRICM101) on TNF-α/IL-1β-induced human lung cells

**DOI:** 10.37796/2211-8039.1378

**Published:** 2022-09-01

**Authors:** Yih-Dih Cheng, Chi-Cheng Lu, Yuan-Man Hsu, Fuu-Jen Tsai, Da-Tian Bau, Shih-Chang Tsai, Ching-Chang Cheng, Jen-Jyh Lin, Yuang-Yu Huang, Yu-Ning Juan, Yu-Jen Chiu, Sheng-Chu Kuo, Jai-Sing Yang, Lii-Tzu Wu

**Affiliations:** a School of Pharmacy, China Medical University, Taichung, 406040, Taiwan; b Department of Pharmacy, China Medical University Hospital, Taichung, 404327, Taiwan; c Department of Sport Performance, National Taiwan University of Sport, Taichung, 404401, Taiwan; d Department of Biological Science and Technology, College of Life Sciences, China Medical University, Taichung, Taiwan, 406040, Taiwan; e Department of Animal Science and Biotechnology, Agriculture College, Tunghai University, Taichung, 407224, Taiwan; f School of Chinese Medicine, College of Chinese Medicine, China Medical University, Taichung, 404333, Taiwan; g China Medical University Children’s Hospital, Taichung, 404327, Taiwan; h Department of Medical Genetics, China Medical University Hospital, Taichung, 404327, Taiwan; i Graduate Institute of Biomedical Sciences, China Medical University, Taichung, 404333, Taiwan; j Terry Fox Cancer Research Laboratory, Department of Medical Research, China Medical University Hospital, Taichung, 404327, Taiwan; k Department of Bioinformatics and Medical Engineering, Asia University, Taichung, 413305, Taiwan; l Department of Biological Science and Technology, China Medical University, Taichung, 406040, Taiwan; m Laboratory Animal Service Center, China Medical University, Taichung, 404333, Taiwan; n Division of Cardiology, Department of Medicine, China Medical University Hospital, Taichung, 404327, Taiwan; o Department of Medical Research, China Medical University Hospital, China Medical University, Taichung, 404327, Taiwan; p Division of Plastic and Reconstructive Surgery, Department of Surgery, Taipei Veterans General Hospital, Taipei, 112201, Taiwan; q Department of Surgery, School of Medicine, National Yang Ming Chiao Tung University, Taipei, 112304, Taiwan; r Institute of Clinical Medicine, National Yang Ming Chiao Tung University, Taipei, 112304, Taiwan; s Department of Microbiology, School of Medicine, China Medical University, Taichung, 404333, Taiwan

**Keywords:** Taiwan chingguan Yihau (NRICM101), Traditional Chinese medicine (TCM), TNF-α/IL-1β-induced lung cell injury, High throughput target screening platform, Next-generation sequencing (NGS)

## Abstract

COVID-19 pandemic has been a global outbreak of coronavirus (SARS-CoV-2 virus) since 2019. Taiwan Chingguan Yihau (NRICM101) is the first traditional Chinese medicine (TCM) classic herbal formula and is widely used for COVID-19 patients in Taiwan and more than 50 nations. This study is to investigate *in silico* target fishing for the components of NRICM101 and to explore whether NRICM101 inhibits cytokines-induced normal human lung cell injury *in vitro*. Our results showed that network prediction of NRICM101 by a high throughput target screening platform showed that NRICM101 has multiple functions that may affect cytokine regulation to prevent human lung cell injury. In addition, NRICM101 revealed protective effects against TNF-α/IL-1β-induced normal human lung HEL 299 cell injury through JNK and p38MAPK kinase signaling. Next-generation sequencing (NGS) analysis of NRICM101 on TNF-α/IL-1β-injured HEL 299 cells indicated that inflammatory pathway, cell movement of macrophages, cellular infiltration by macrophages, and Th1/Th2 immuno-regulation pathways were included. Thus, NRICM101 is a therapeutic agent, and it can improve COVID-19 syndrome to confer beneficial effects through multiple targeting and multiple mechanisms.

## 1. Introduction

**Fig. 1:**
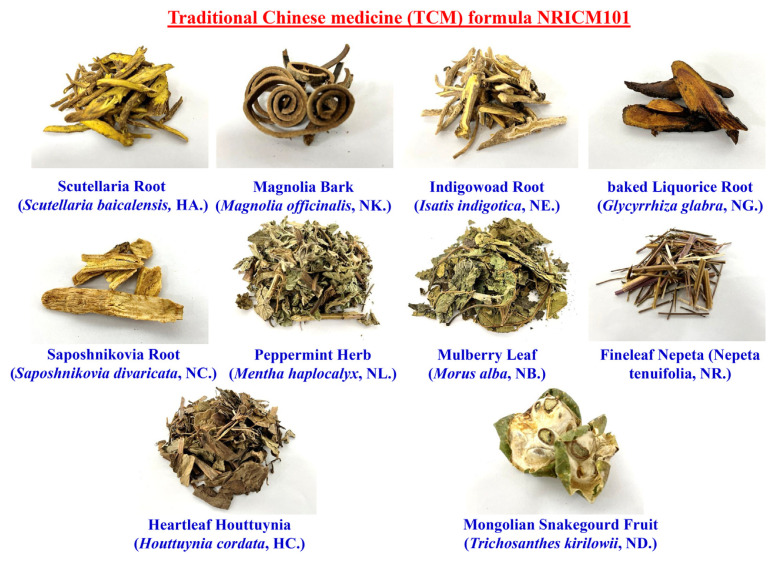
The components of traditional Chinese medicine (TCM) formula NRICM101.

A traditional Chinese medicine formula Taiwan Chingguan Yihau (NRICM101) is the first traditional Chinese medicine (TCM) classic herbal formula that is widely used in more than 50 nations for COVID-19 patients [1]. NRICM101 consists of ten herbs (Fig. 1), including Scutellaria Root (*Scutellaria baicalensis*, HA.), Mongolian Snakegourd Fruit (*Trichosanthes kirilowii*, ND.), Indigowoad Root (*Isatis indigotica*, NE.), baked Liquorice Root (*Glycyrrhiza glabra*, NG.), Saposhnikovia Root (*Saposhnikovia divaricata*, NC.), Peppermint Herb (*Mentha haplocalyx*, NL.), Mulberry Leaf (*Morus alba*, NB.), Fineleaf Nepeta (*Nepeta tenuifolia*, NR.), Heartleaf Houttuynia (*Houttuynia cordata*, HC.), and Magnolia Bark (*Magnolia officinalis*, NK.) [1]. All herbal preparations have been shown to exert potential for inhibiting SARS-CoV-2 of respiratory infection and immune-modulatory effect, indicated by the National Research Institute of Chinese Medicine (NRICM) of Taiwan after evaluating clinical symptoms. Scientific evidence based on a clinical perspective indicates that NRICM101 may disrupt COVID-19 disease progression via antiviral and anti-inflammatory effects to offer promise as a multi-target agent for the prevention and treatment [1]. NRICM101 is an anti-SARS-CoV-2 therapeutic agent as mentioned in COVID-19 treatment guidelines in Taiwan (https://www.mohw.gov.tw/cp-16-60830-1.html). Patients with severe COVID-19 to cause a large number of pro-inflammatory macrophages were found in bronchoalveolar lavage fluid and released a large number of IL-1, TNF-α, IL-6 IL-8, CSF, and MCP-1 to induce cytokine storm [2]. Therapeutics with the potential to mitigate inflammatory cytokines may attenuate disease progression and mortality [3–5].

The manifestations of SARS-CoV-2 infection vary widely from asymptomatic diseases to severe pneumonia and life-threatening complications [6,7]. Abnormal lung function is the most common feature among patients of SARS-CoV-2 infection and can be complicated by acute respiratory distress syndrome (ARDS), particularly in elderly people with multiple comorbidities [8–10]. As the pandemic of COVID-19 continues which are with high levels of inflammatory markers, and are often accompanied by evidence of pulmonary fibrosis including interstitial thickening, coarse reticular patterns, and parenchymal bands lymphopenia [11–13]. Retrospective analyses have found that elevated inflammatory markers (such as erythrocyte sedimentation rate, C-reactive protein, ferritin, TNF-α, IL-1, and IL-6) are higher in patients who died compared to survivors [14,15]. Cytokine-release syndrome (CRS), cytokine storm and post-COVID syndrome (PCS) are suggested as two of the major pathophysiological processes of SARS-CoV-2 infection and promote the deterioration of COVID-19 [8–10]. Laboratory analysis demonstrated that SARS-CoV-2 spike (S) protein was associated with the up-regulation of AT1 signaling which led to the induction of transcriptional regulatory molecules (NF-κB, c-Fos, and MAPK activation) [16]. The signal transduction may lead to secrete high levels of inflammatory cytokines that are observed in the lungs of COVID-19 patients like cytokine storm and pulmonary fibrosis due to macrophage activation and infiltration [17,18].

**Fig. 2:**
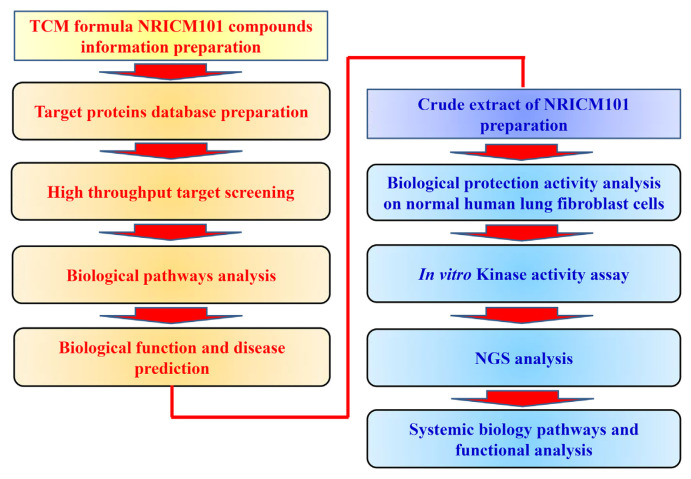
Study design and flowchart of NRICM101 via in silico and in vitro studies.

Our goal of this study is to understand whether NRICM101 could interact with other target proteins and inhibit cytokine-induced human lung cell injury. The study design and schematics were performed by *in silico* target fishing for the components of NRICM101, the workflow of *in silico* assay and *in vitro* bioactivity analysis are presented in Fig. 2.

## 2. Materials and methods

### 2.1. In silico studies of high throughput target screening and network analysis

The major components of NRICM101 (Baicalein, Baicalin, Wogonin, Wogonoside, Decanoyl acetaldehyde, Lauric aldehyde, Quercetin, Linalool, Luteolin, Kaempferol, N-Methyl-1-deoxynojirimycin, 2-O-α-d-galactopyranosyl-deoxy-nojirimycin, fagomine, Rutin, Isoquercitrin, 3-O-glucuronide, β-sitosterol, Stigmasterol, Camposterol, 5-O-methylvisammioside, prim-O-glucosylcimifugin, Cimifugin, sec-O-glucosylhamaudol, Hamaudol, Lignoceric acid, Dacursin, Bryonolic acid, Cucurbitacin B, Cucurbitacin D, 23′24-dihydrocucurbitacin B, Epigoitrin, Indigotin, Indirubin, Clionasterol, Sinigrin, Indoxyl β-d-glucoside, Epigoitrin, Palmitic acid, Adenosine, Glycyrrhetic acid, Glycyrrhizic acid, Glabrolide, Liquiritin, liquiritingenin, isoliquiritin, iso-liquiritingenin, Magnolol, Honokiol, α-eudesmol, β-eudesmol, Menthol, Menthone, Glucoside, Apigenin, Chlorogenic acid, Caryophyllene, Pulegone, Menthone, α-Phytosterol, α-Tocopherolquinone) were sketched using BIOVIA Draw and prepared for generating the fitting compound for protonated iso-mers and tautomers at pH 7.4. A total of 16,035 target proteins by pharmacophore models in PharmaDB (BIOVIA Discovery Studio 2020 software; Dassault Systèmes) were then applied as screening targets for the 60 components of NRICM101 [19,20]. For network analysis, a goodness-of-fit value of >0.6 was considered to be potential compound target proteins. To generate a corresponding molecular network of those target proteins, all human target genes were set as focus molecules and analyzed using a core analysis tool in IPA (IPA 2020; Qiagen Sciences, Inc.). All presented pathways were deemed to be statistically significant according to Fisher’s exact t-test (P< 0.05) [12].

### 2.2. NRICM101 crude extracts preparation

NRICM101 crude extracts were obtained from Department of Pharmacy, China Medical University Hospital and prepared with the following composition: Scutellaria Root (*S. baicalensis*, HA, 18.75 g), Heartleaf Houttuynia (*H. cordata*, HC, 18.75 g), Mulberry Leaf (*M. alba*, NB, 11.25 g), Saposhnikovia Root (*S. divaricata*, NC, 7.50 g), Mongolian Snakegourd Fruit (*T. kirilowii*, ND, 18.75 g), Indigowoad Root (*I. indigotica*, NE, 18.75 g), baked Liquorice Root (*G. glabra*, NG, 7.50 g), Magnolia Bark (*M. officinalis*, NK, 11.25 g), Peppermint Herb (*M. haplocalyx*, NL, 11.25 g), and Fineleaf Nepeta (*N. tenuifolia*, NR, 11.25 g). For a patient’s daily dose, a full set of herbs and 1 L of water were placed in a boiler, boiled, and simmered for the decoction to reduce to 300 mL. The NRICM101 formulation was boiled with 1000 mL distilled water for 60 min into a 350 mL decoction and then concentrated under reduced pressure to 7.17 g by Rotary Evaporator (N–1300VF/OSB-2200; EYELA, Japan).

### 2.3. Cell viability and cell morphology detection

Normal human embryonic lung fibroblast cell line (HEL 299) was obtained from the Bioresources Collection and Research Center (cat. no. 60117) and Food Industry Research and Development Institute (Hsinchu, Taiwan). HEL 299 cells were cultured in Dulbecco’s modified Eagle’s medium (DMEM) with 2 mM l-glutamine, 10% fetal bovine serum (FBS), 100 U/mL penicillin, and 100 μg/mL streptomycin (Life Technologies) in 75-T culture flasks under a humidified atmosphere with 5% CO_2_ at 37 °C. HEL 299 cells were cultured in 24-well plates at 2.5 × 10^5^ cells/mL/well. The cells were treated with TNF-α (50 ng/mL) and IL-1β (50 ng/mL) (Sigma–Aldrich and Merck KGaA), and NRICM101 (50 and 100 μg/mL) for 24 h. Cell viability was detected by 3-(4,5-Dimethylthiazol-2-yl)-2,5-diphenyltetrazolium bromide (MTT) (Sigma–Aldrich and Merck KGaA) as previously described [21,22]. Blue MTT formazan crystals were dissolved in DMSO and measured using an ELISA reader at 570 nm [21,22]. To determine the cell morphological changes in cytokines-induced normal human lung cell injury, HEL 299 cells were treated with TNF-α, IL-1β (50 ng/mL, individual), and NRICM101 (50 and 100 μg/mL) for 24 h. Cell morphology was examined by phase-contrast microscopy (Leica Microsystems GmbH; magnification, 200X) [23].

### 2.4. Cell injury and DNA condensation by DAPI stain

HEL 299 cells were treated with TNF-α, IL-1β (50 ng/mL, individual), and NRICM101 (50 and 100 μg/mL) for 24 h. Cell injury and DNA condensation were examined by DAPI staining (Sigma–Aldrich and Merck KGaA). The cell fluorescent imaging was examined by Echo Revolve microscope (Echo Laboratories, San Diego, California, 200X) [24,25].

### 2.5. JNK and p38MAPK kinase activities assay

JNK and p38MAPK kinase activities assay were examined by phosphorylated protein kinase sandwich ELISA assay. p-p38 MAPK (Thr180/Tyr182) (cat. no. 7946), p-JNK (Thr183/Tyr185) (cat. no. #7325), and assays were performed according to the manufacturer’s protocols (PathScan Sandwich ELISA kits; Cell Signaling Technology, Inc.). HEL 299 cells were treated with TNF-α, IL-1β (50 ng/mL, individual), and NRICM101 (50 and 100 μg/mL) for 12 h. Cells were harvested and total proteins were collected. Proteins were incubated in appropriate antibody-coated micro-wells overnight at 4 °C. The 100 μL/well of the appropriate antibody was added for 1.5 h at 37 °C, and an HRP-linked secondary antibody was added for 60 min at 37 °C. Absorbance was measured by an ELISA reader (Anthos 2001) at 450 nm as previously described [26,27].

### 2.6. Next-generation sequencing (RNA sequencing transcriptional profile) analysis by whole transcriptome sequencing

HEL 299 cells were treated with TNF-α, IL-1β (50 ng/mL, individual), and NRICM101 (100 μg/mL) for 12 h. Total RNA was extracted using TRIzol ® reagent (Invitrogen, USA). Purified RNA was using a Bioanalyzer 2100 (Agilent Technology, USA) with an RNA 6000 LabChip Kit (Agilent Technology, USA) and measured at OD_260_ nm by ND-1000 spectrophotometer (Nanodrop Technology, USA). RNA sample preparation procedures were performed according to Illumina’s official standard protocol. For library construction, the SureSelect XT HS2 mRNA Library Preparation Kit (Agilent, USA) was used, followed by AMPure XP beads (Beckman Coulter, USA). The Illumina sequencing-by-synthesis technology (Illumina, USA) (300-cycle paired-end read; 150 PE) was used for RNA sequencing. Sequencing results analysis (FASTQ reads) were using Illumina’s base-calling program bcl2fastq v2.20. Adaptor clipping and sequence quality trimming were done using Trimmomatic v0.36. HISAT2 for RNA alignment and the expression levels were normalized by calculating transcripts per million mapped reads. Differentially expressed genes between TNF-α, IL-1β (50 ng/mL, individual), and NRICM101 (100 μg/mL)-exposed and control groups [TNF-α, IL-1β (50 ng/mL, individual)] were selected using three criteria: P < 0.005, adj P < 0.05 and absolute fold change of ≥1.5). The differentially expressed genes were selected by StringTie (StringTir v2.1.4) and DEseq (DEseq v1.39.0) with genome bias detection/correction using Welgene Biotech’s in-house pipeline. The p-value was calculated using the hypergeometric p-value calculated as the probability of random drawing [28,29].

### 2.7. Network and signaling pathways analysis by ingenuity pathway analysis (IPA) and Kyoto Encyclopedia of Genes and Genomes (KEGG)

For network and signaling pathways analysis of the whole transcriptome sequencing database, a total of 331 human target genes were prepared and subsequently analyzed using a core analysis tool in IPA software (IPA 2021; Qiagen Sciences, Inc.) and Kyoto Encyclopedia of Genes and Genomes (KEGG) database. The network analysis rankings were calculated based on statistical significance using Fisher’s exact *t*-test (p < 0.05) [28,29].

### 2.8. Statistical analysis

For each *in vitro* study, three independent experiments were conducted. Data are presented as the mean ± standard (SD) deviation. One-way analysis of variance followed by Dunnett’s test and Tukey’s post hoc test was conducted to analyze the differences between two groups and among multiple groups by SPSS software version 25.0 (IBM, Corp.). ***P < 0.001 and ###P < 0.001 were considered to indicate a statistically significant difference.

## 3. Results

### 3.1. In silico study

#### 3.1.1. Pathways and network prediction of NRICM101 by high throughput target screening platform

**Fig. 3:**
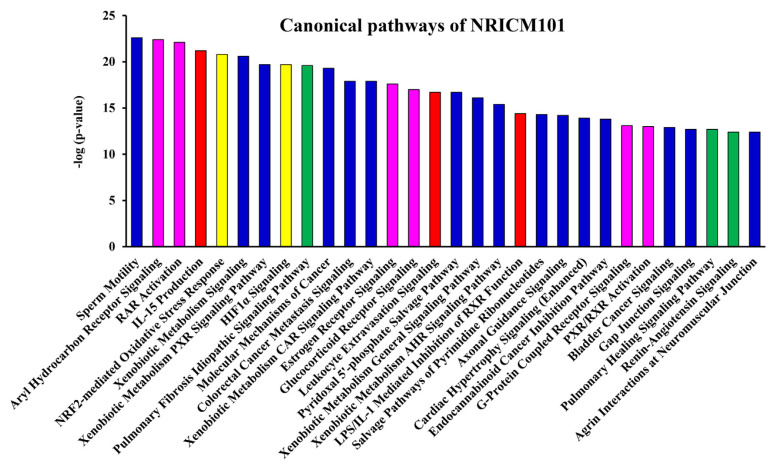
Canonical pathways of NRICM101 via high throughput target screening platform analysis.

**Fig. 4:**
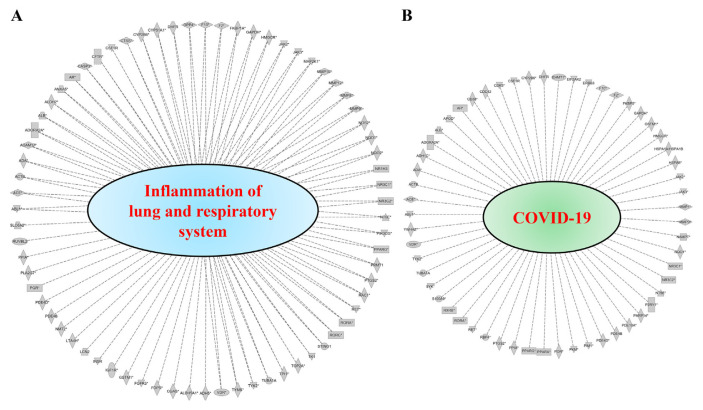
Analysis of NRICM101 on molecule targets contribute to inflammation of the lung and respiratory system (A) and COVID-19 (B).

**Fig. 5:**
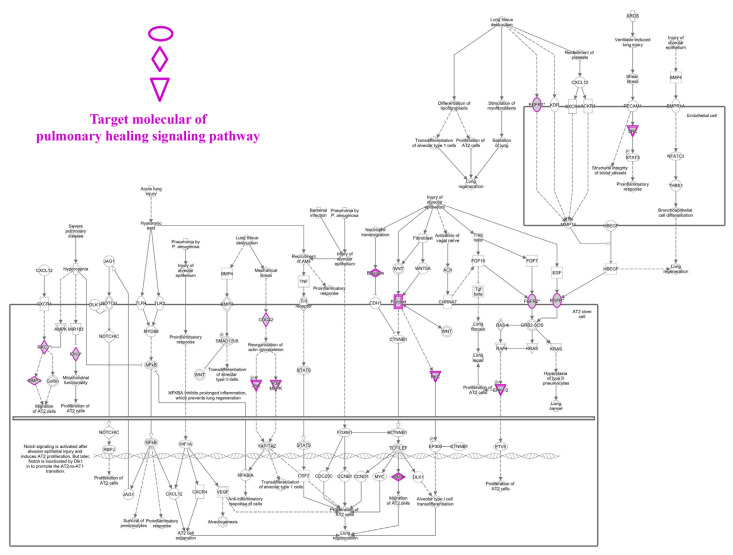
Network analysis of target molecules of pulmonary healing signaling pathways.

**Fig. 6:**
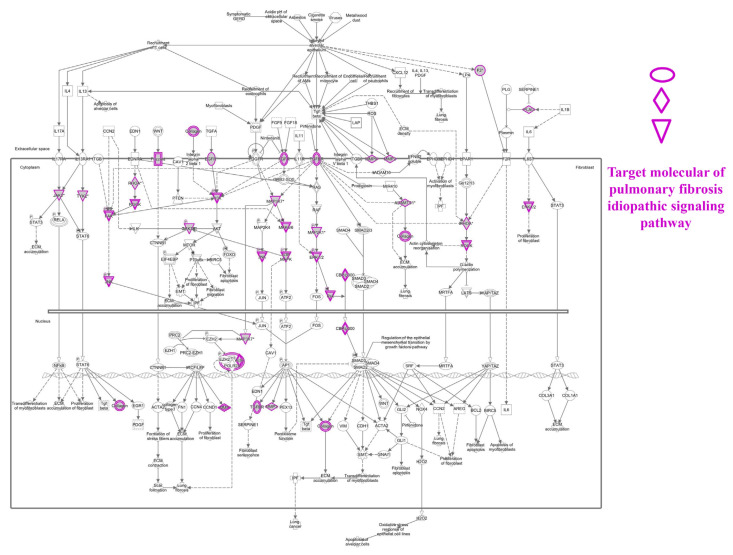
Target molecular of pulmonary fibrosis idiopathic signaling pathway via network analysis.

**Fig. 7:**
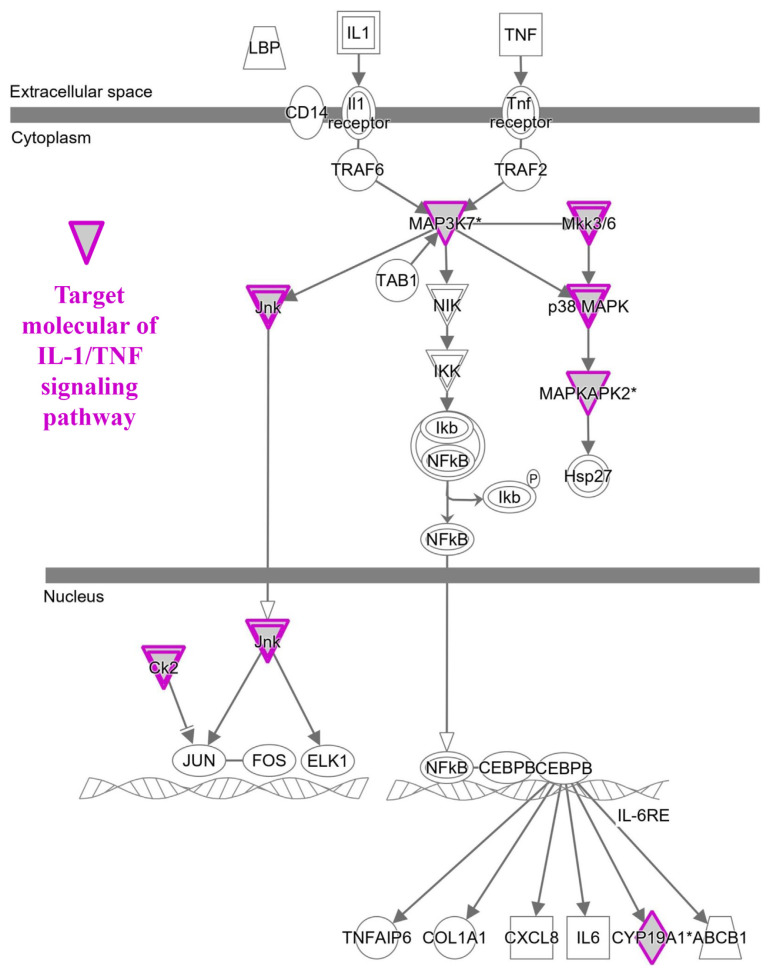
Pathway analysis with target molecular of IL-1/TNF signaling.

To predict target proteins of NRICM101, we used high throughput target screening platform analysis. The canonical pathway of NRICM101 was showed in Fig. 3, bars correspond to the related pathways associated with NRICM101 include five part: cytokines and immune cells (IL-15 Production, Leukocyte Extravasation Signaling, LPS/IL-1 Mediated Inhibition of RXR Function; red lines), oxidative stress and antioxidant (NRF2-mediated Oxidative Stress Response, HIF1α Signaling; yellow lines), pulmonary related disease (Pulmonary Fibrosis Idiopathic Signaling Pathway, Pulmonary Healing Signaling Pathway, Renin-Angiotensin Signaling; green lines), receptor (Aryl Hydrocarbon Receptor Signaling, RAR Activation, Estrogen Receptor Signaling, Glucocorticoid Receptor Signaling, G-Protein Coupled Receptor Signaling, PXR/RXR Activation; purple lines) and others (Sperm Motility, Xenobiotic Metabolism Signaling, Xenobiotic Metabolism PXR Signaling Pathway, Molecular Mechanisms of Cancer, Colorectal Cancer Metastasis Signaling, Xenobiotic Metabolism CAR Signaling Pathway, Pyridoxal 5′-phosphate Salvage Pathway, Xenobiotic Metabolism General Signaling Pathway, Xenobiotic Metabolism AHR Signaling Pathway, Salvage Pathways of Pyrimidine Ribonucleotides, Axonal Guidance Signaling, Cardiac Hypertrophy Signaling (Enhanced), Endocannabinoid Cancer Inhibition Pathway, Bladder Cancer Signaling, Gap Junction Signaling, Agrin Interactions at Neuromuscular Junction; blue lines). We summarized that molecule targets contribute to inflammation of the lung and respiratory systems (Fig. 4A) and COVID-19 (Fig. 4B) on NRICM101. In addition, network analysis and visualization revealed pulmonary healing signaling pathways (Fig. 5), pulmonary fibrosis idiopathic (Fig. 6), and IL-1/TNF signaling pathway (Fig. 7) with a significant positive correlation. Our results suggest that NRICM101 has not only anti-SARS-CoV-2 activity but also pneumonic protection and immune-regulated effects. Here, it is hypothesized that NRICM101 has multiple functions that may affect cytokine regulation and prevent human lung cell injury.

### 3.2. In vitro study

#### 3.2.1. NRICM101 revealed protective effects against TNF-α/IL-1β-induced normal human lung HEL 299 cell injury

**Fig. 8:**
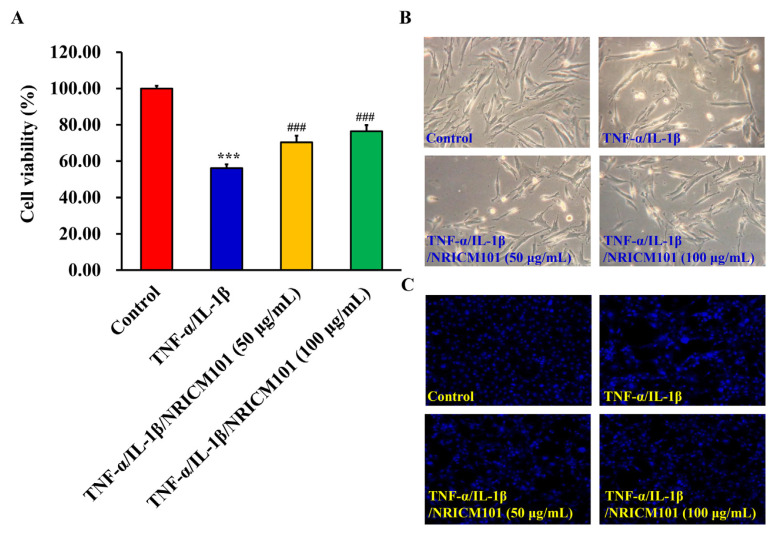
Effect of cell viability on TNF-α/IL-1β and NRICM101 co-incubation in HEL 299 cells. The cells (2.5 × 10^5^ cells/mL/well) were exposed to TNF-α/IL-1β and NRICM101 (50 and 100 μg/mL) for 24 h. (A) Cell viability was detected by the MTT assay. Data are presented as the mean ± standard deviation (n = 3) and analyzed using one-way ANOVA followed by Tukey’s post hoc test. ***P < 0.001 vs control; ###P < 0.001 vs TNF-α/IL-1β group. (B) Cell morphology was examined by phase-contrast microscopy. (C) Cell injury and DNA condensation were evaluated via DAPI stain.

To confirm the results of *in silico* prediction, we further investigated the potential protective effects of NRICM101 on TNF-α/IL-1β-induced cell injury in HEL 299 cells. The results demonstrated that cell viability significantly decreased in TNF-α/IL-1β (50 ng/mL, individual) treatment (Fig. 8A) and increased dead cells (Fig. 8B) and DNA condensation (Fig. 8C). However, the treatment with TNF-α/IL-1β (50 ng/mL, individual) and NRICM101 (50 and 100 μg/mL) showed increased viable cells (Fig. 8A) and decreased dead cells (Fig. 8B) and DNA condensation (Fig. 8C). These results suggest that NRICM101 might exert protective effects against TNF-α/IL-1β-induced cell injury in HEL 299 cells.

#### 3.2.2. NRICM101 against TNF-α/IL-1β-induced HEL 299 cell injury was attenuated through JNK and p38MAPK kinase activities

**Fig. 9:**
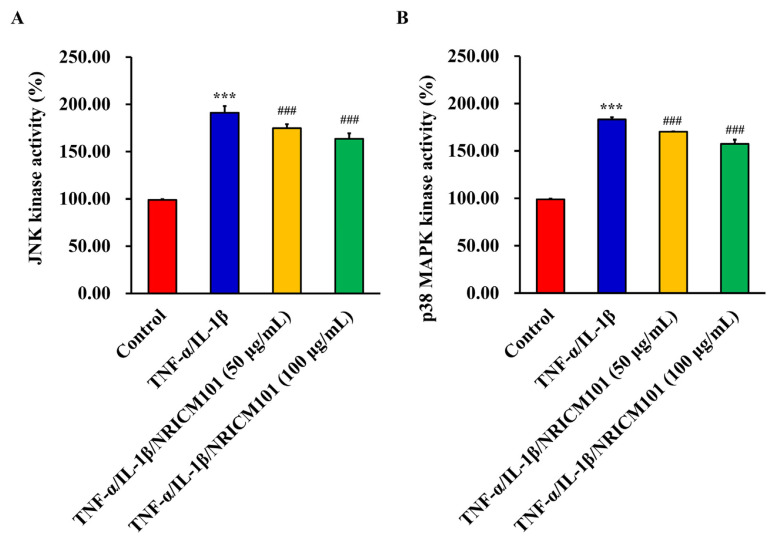
Effect of JNK and p38 MAPK kinase activities on HEL 299 cells after TNF-α/IL-1β and NRICM101 exposure. HEL 299 cells (2.5 × 10^5^ cells/mL/well) were treated with TNF-α/IL-1β and NRICM101 (50 and 100 μg/mL) for 24 h. (A) JNK and (B) p38MAPK kinase activities assay were examined by phosphorylated protein kinase sandwich ELISA assay. Data are presented as the mean ± standard deviation (n = 3) and analyzed using one-way ANOVA followed by Tukey’s post hoc test. ***P < 0.001 vs control; ###P < 0.001 vs TNF-α/IL-1β group.

In order to further confirm the results of TNF-α/IL-1β-induced cell injury, both of JNK and p38MAPK kinase activity assays were examined in TNF-α/IL-1β-treated HEL 299 cells after NRICM101 treatment. Our results in Fig. 9 showed that phosphorylation of JNK and p38MAPK significantly increased in TNF-α/IL-1β treatment, and NRICM101 significantly attenuated JNK and p38MAPK phosphorylation on TNF-α/IL-1β-treated HEL 299 cells. The abovementioned findings revealed the concentration-dependent effects of NRICM101 on the down-regulation of JNK and p38MAPK kinase in TNF-α/IL-1β-treated HEL 299 cells.

#### 3.2.3. Next-generation sequencing analysis of NRICM101 on TNF-α/IL-1β-injured HEL 299 cells

**Fig. 10:**
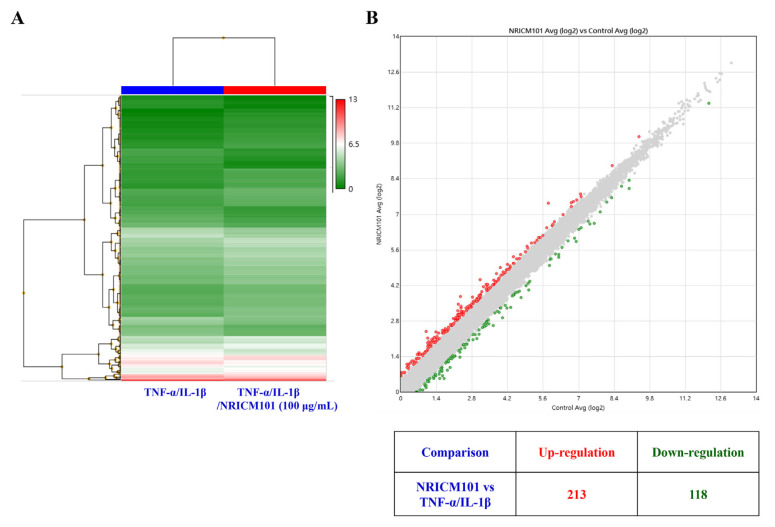
RNA sequencing transcriptional profile of the (A) two samples [HEL 299 cells before TNF-α/IL-1β and TNF-α/IL-1β/NRICM101 (100 μg/mL) treatment]. (B) Differential expression of MA plot was performed and showed that red dots (213) represent upregulated and green dots (118) downregulated between the TNF-α/IL-1β and TNF-α/IL-1β/NRICM101 groups.

**Fig. 11:**
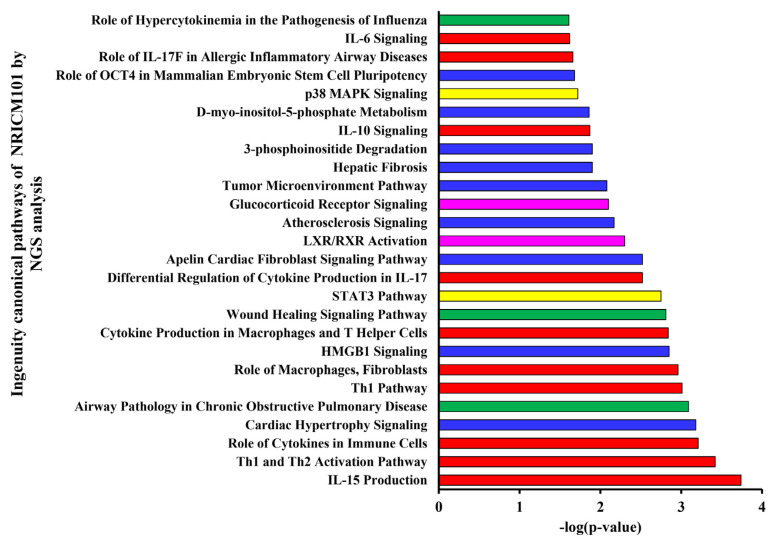
Ingenuity canonical pathways of NRICM101 by NGS analysis. The most significantly enriched pathway was shown and involved in IL-15 production.

**Fig. 12:**
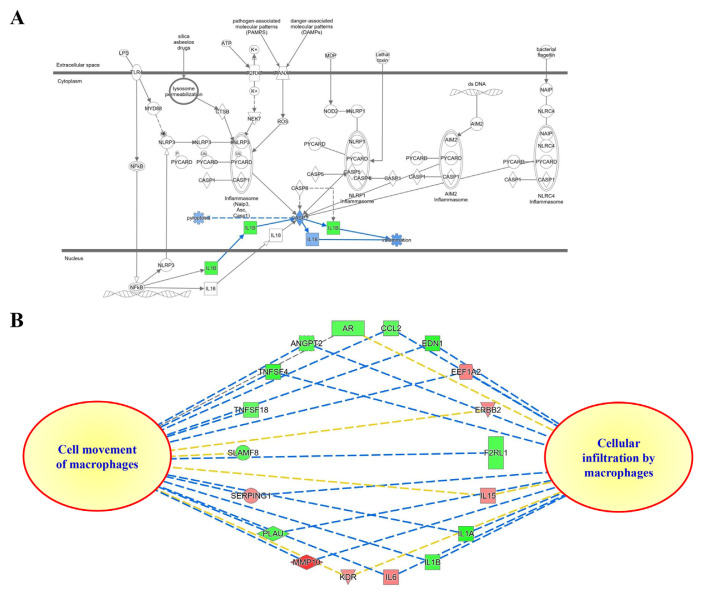
(A) Predictive target genes and associated IL-1B cytokine. (B) IPA core analysis of potential targets of NRICM101 in cell movement and cellular infiltration by macrophages.

**Fig. 13:**
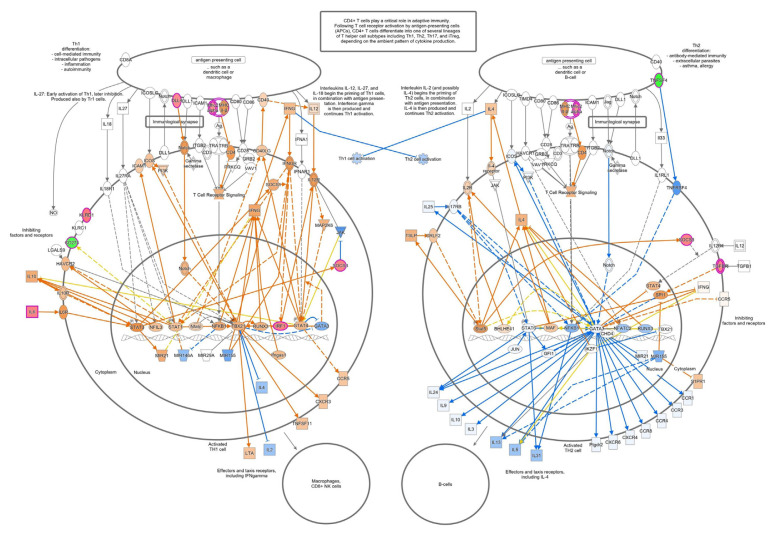
Predictive target genes and associated Th1/Th2 immuno-regulation pathways in the pulmonary system.

To improve insight into the biological activity of NRICM101 in TNF-α/IL-1β-induced cell injury in HEL 299 cells, RNA sequencing transcriptional profile analysis was performed. As shown in Fig. 10A, normalized RNA-sequencing data from NRICM101-treated samples and the control group (TNF-α/IL-1β) were clustered, indicating a significantly different gene expression analysis. In Fig. 10B, red dots mean significantly up-regulated genes, and green dots mean significantly down-regulated genes in MA plot. The 213 genes were up-regulated and 118 genes were down-regulated. Supplementary Table S1 (https://www.biomedicinej.com/cgi/editor.cgi?article=1378&window=additional_files&context=biomedicine) showed the raw sequencing data of NRICM101-treated TNF-α/IL-1β-injured HEL 299 cells. To further determine the mechanism of action (MOA) of the genes and associated functions, the Ingenuity pathway analysis (IPA) database and KEGG database were used. The most significantly enriched pathways were selected and shown in Fig. 11. The pathway included IL-15 Production, Th1 and Th2 Activation Pathway, Role of Cytokines in Immune Cells, Cardiac Hypertrophy Signaling, Airway Pathology in Chronic Obstructive Pulmonary Disease, Th1 Pathway, Role of Macrophages, Fibroblasts, HMGB1 Signaling, Cytokine Production in Macrophages and T Helper Cells, Wound Healing Signaling Pathway, STAT3 Pathway, Differential Regulation of Cytokine Production in IL-17, Apelin Cardiac Fibroblast Signaling Pathway, LXR/RXR Activation, Atherosclerosis Signaling, Glucocorticoid Receptor Signaling, Tumor Microenvironment Pathway, Hepatic Fibrosis, 3-phosphoinositide Degradation, IL-10 Signaling, D-myo-inositol-5-phosphate Metabolism, p38 MAPK Signaling, Role of OCT4 in Mammalian Embryonic Stem Cell Pluripotency, Role of IL-17F in Allergic Inflammatory Airway Diseases, IL-6 Signaling, Role of Hypercytokinemia in the Pathogenesis of Influenza. Our results showed the IL-1A, IL-1B, TNFSF4, TNFSF18, IFI44L, IL32, and CCL2 genes were down-regulated of NRICM101 on TNF-α/IL-1β-injured HEL 299 cells (Fig. 12 and Supplementary Table S1 (https://www.biomedicinej.com/cgi/editor.cgi?article=1378&window=additional_files&context=biomedicine)). In addition, those results suggest NRICM101 regulated TNF-α/IL-1β medicated inflammatory pathway (Fig. 12A), cell movement of macrophages, cellular infiltration by macrophages (Fig. 12B), and Th1/Th2 immunoregulation pathways (Fig. 13) in TNF-α/IL-1β-injured HEL 299 cells.

## 4. Discussion

Traditional Chinese medicine (TCM) formula, Taiwan Chingguan Yihau (NRICM101), has been administered orally to COVID-19 patients in Taiwan [1]. NRICM101 consisted of ten herbs [1], and it was demonstrated that Scutellaria Root (*S. baicalensis*, HA.), Heartleaf Houttuynia (*H. cordata*, HC.) and Peppermint Herb (*M. haplocalyx*, NL.) potentially blocked spike (S) protein of SARS-CoV-2 and host’s angiotensin-converting enzyme 2 (ACE2) interaction [30–34]. In addition, Scutellaria Root (*S. baicalensis*, HA.), Peppermint Herb (*M. haplocalyx*, NL.), Fineleaf Nepeta (*N. tenuifolia*, NR.), Magnolia Bark (*M. officinalis*, NK.) and Mulberry Leaf (*M. alba*, NB.) inhibited 3C-like protease (3CL^pro^) or main protease (M^pro^) activity of SARS-CoV-2 [34–41]. In our early study demonstrated that baicalin, one of the active components in Scutellaria Root (*S. baicalensis*, HA.), has a high binding affinity and inhibitory effect on Papain-like Protease (PL^pro^) [9,19]. *In silico* study showed quercetin and kaempferol of Heartleaf Houttuynia (*H. cordata*, HC.) had high binding affinities for 3C-like protease (3CL^pro^) and RNA-dependent RNA polymerase (RDRP) of SARS-CoV-2 [19]. Honokiol, isolated from Magnolia Bark (*M. officinalis*, NK.), exerted a high binding affinity on angiotensin-converting enzyme 2 (ACE2) [38]. Sinigrin and hesperetin, two active components in Indigowoad Root (*I. indigotica*, NE.), had high binding affinities for 3C-like protease (3CL^pro^) of SARS-CoV-2 [42,43]. The mechanism studies on pharmacological activities of NRICM101 in the treatment of COVID-19 are limited to confirming the interaction of the formula with viral proteins and other structures according to identified pathogenic pathways of these remedies must be performed before further clinical trials. In our study, the results of NGS analysis indicated NRICM101 regulated SARS-CoV-2 infected pathways on COVID-19 (Supplementary Figure S1 (https://www.biomedicinej.com/cgi/editor.cgi?article=1378&window=additional_files&context=biomedicine)). The experimental studies showed that all active components of NRICM101 may be a potent anti-SARS-CoV-2 agent for COVID-19.

It has been demonstrated that an association between poor outcome and cytokine-release syndrome (CRS) of COVID-19 [44,45]. In this study, we first analyze target proteins, biological pathways, disease, and function of bioactive compounds of NRICM101 using a high throughput target screening system. According in silico study results, we predicted that NRICM101 exerted multiple targets and signaling pathways (Fig. 3). Further analysis revealed that the main bioactive compounds contained within the cytokine-related pathways (IL-15 production, LPS/IL-1 Mediated Inhibition of RXR Function) (Fig. 7), oxidative stress response pathways (NRF2-mediated Oxidative Stress Response, HIF1α Signaling), pulmonary related signaling pathways (Pulmonary Fibrosis Idiopathic Signaling Pathway, Pulmonary Healing Signaling Pathway) (Figs. 4–6). NRICM101 potentially affected the inflammatory cytokine signaling, including TNFR2, TNFR1, IL-1, IL-2, IL-4, IL-6, IL-7, IL-12, IL-13, IL-17, and IL-23 Signaling (Fig. 3), all of which were previously identified in COVID-19 [46,47]. Through high throughput target screening and signaling pathway analysis on the inflammation of the lung, respiratory systems and SARS-CoV-2, these compounds contained within NRICM101 not only serve important roles on anti-SARS-CoV-2 agents but can also provide potential points for developing lung and respiratory system protection agents. The importance of nodes, the results in Fig. 7 found the MAP3K7, JNK, MKK3/6, p38MAPK, and MAPKAPK2 target proteins were involved in IL-1/TNF signaling pathways.

The first purpose of this study was to investigate the effects of NRICM101 on TNF-α/IL-1β-induced normal human lung HEL 299 cell injury. Our results demonstrated the protective effects of NRICM101 against TNF-α/IL-1-induced injury (Fig. 8A), cytotoxicity (Fig. 8B), DNA condensation and damage (Fig. 8C), and JNK (Fig. 9A), p38MAPK kinase activities (Fig. 9B). Second, NGS analysis was conducted to investigate the molecular mechanisms and signaling pathway of NRICM101 on TNF-α/IL-1β-injured HEL 299 cells. Results for Fig. 10 and Supplementary Table S1 (https://www.biomedicinej.com/cgi/editor.cgi?article=1378&window=additional_files&context=biomedicine) showed a total of 213 genes were upregulated, and 118 genes were down-regulated. A network of the associations of different genes was generated following IPA analysis, as shown in Fig. 11, Which regulation of cytokines and immune cells (IL-15 production, Th1 and Th2 activation pathway, role of cytokines in immune cells, Th1 pathway, role of macrophages and fibroblasts, cytokine production in macrophages and T helper cells, differential regulation of cytokine production in IL-17, IL-10 signaling, role of IL-17F in allergic inflammatory airway diseases, IL-6 signaling; red lines), pulmonary related disease (airway pathology in chronic obstructive pulmonary disease, role of hypercytokinemia in the pathogenesis of influenza, wound healing signaling pathway; green lines), kinase (STAT3 pathway, p38 MAPK signaling; yellow lines), receptor (LXR/RXR activation, glucocorticoid receptor signaling; purple lines) and others (cardiac hypertrophy signaling, HMGB1 signaling, apelin cardiac fibroblast signaling pathway, atherosclerosis signaling, tumor microenvironment pathway, hepatic fibrosis, 3-phosphoinositide degradation, dmyo-inositol-5-phosphate metabolism, role of oct4 in mammalian embryonic stem cell pluripotency; blue lines) played main roles of NRICM101 on TNF-α/IL-1β-injured HEL 299 cells. These results suggest the importance of the immune regulation of NRICM101. In Fig. 11, the ingenuity canonical pathways and major target proteins of NRICM101 were shown, including IL-1-mediated inflammatory signaling (Fig. 12A), cell movement of macrophages, cellular infiltration by macrophages (Fig. 12B) and Th1 and Th2 activation signaling (Fig. 13). Surprisingly, the results of NGS analysis demonstrated that IL-1A, IL-1B, TNFSF4, TNFSF18, IFI44L, IL32, and CCL2 genes were down-regulated of NRICM101 on TNF-α/IL-1β-injured HEL 299 cells (Supplementary Table S1 (https://www.biomedicinej.com/cgi/editor.cgi?article=1378&window=additional_files&context=biomedicine)). In the present study, *in vitro* study results are not only support our *in silico* results but also suggest a protective role and immuneregulation function of the NRICM101 against normal human lung cell injury.

IL-15 is an immune-regulatory cytokine and plays an important role in anti-viral properties [48]. IL-15 is a T cell response to cytokine and is expressed in myeloid cells. IL-15 activates natural killer (NK) cells and then modulates inflammation when virus-infect host cells [48,49]. It has been reported that IL-15 expression increases innate immune responses through the induction of NK cells and CD^8+^ T cells, and then decreased IL-4, IL-5, and IL-13. IL-15 inhibits viral replication, reduces viral loads and reduces SARS-CoV-2-induced inflammation and fibrosis [48–50]. Our *in silico* and *in vitro* studies showed that IL-15 production signaling is involved in TNF-α/IL-1β-induced normal human lung cell injury after NRICM101 treatment. Our results suggest that NRICM101 may affect T cell proliferation, activation, and NK cells activation. In future experiments, we will focus on IL-15 production signaling and design a series *in vivo* experiments of functional assays to include T cell proliferation, T cell activation, and NK cell activation and Th1, Th2 regulation.

Previous studies demonstrated that baicalin and baicalein in Scutellaria Root (*S. baicalensis*, HA.) can attenuate cytokine-induced and chemokine-induced inflammation [9,19]. Syringic, vanillic, p-hydroxybenzoic and ferulic acids of Heartleaf Houttuynia (*H. cordata*, HC.) and Liquorice (*G. glabra*, NG.) extract, glycyrrhizin suppressed lipopolysaccharide (LPS)-stimulated expression of PGE2, iNOS, IL-1β, TNF-α and IL-6 levels in LPS-induced RAW264.7 inflammatory models and LPS-induced inflammatory in endometrial epithelial cells [51–53]. *In vitro* study showed that IL-1β-induced activation of inflammatory factors (TNF-α, IL-6, INOS, and COX2) was suppressed by Mulberry Leaf (*M. alba*, NB.) [54]. 18β-Glycyrrhetinic acid of Liquorice (*G. glabra*, NG.) inhibits IL-1β-induced inflammatory response in mouse chondrocytes [55]. In addition, Licochalcone A of Liquorice (*G. glabra*, NG.) attenuates LPS-induced acute kidney injury [56]. Our findings of the present study were consistent with previous study results on the protective effect of NRICM101 in TNF-α/IL-1β-injured HEL 299 cells.

## 5. Conclusion

Although most of the bioactive components of NRICM101 were selected for this study, the list of compounds in Table 1 was investigated to be not representative of all the chemical components in NRICM101. Finally, our study had several limitations in this study. All *in silico* and *in vitro* approaches require further *in vivo* and clinical experimental verification. Collectively, our results revealed the potential of NRICM101 as a therapeutic agent based on TCM that may confer beneficial effects on COVID-19 patients through multiple targeting and multiple mechanisms. Furthermore, the major bioactive compounds of NRICM101 are worthy of attention as striking candidates for anti-viral agent and protective agent discovery studies on COVID-19. It may be useful for further studies on the therapeutic properties of NRICM101 and its constituents on a molecular level, with the aim of findings that might contribute to improving care for COVID-19 patients.

## Supplementary Information



## Figures and Tables

**Table 1:** The components of NRICM101.

Scientific Name	Compound Name
Scutellaria Root *(Scutellaria baicalensis)*	Baicalein
	Baicalin
	Wogonin
	Wogonoside
Heartleaf Houttuynia (*Houttuynia cordata*)	Decanoyl acetaldehyde
	Lauric aldehyde
	Quercetin
	Linalool
	Luteolin
	Kaempferol
Mulberry Leaf (*Morus alba*)	N-Methyl-1-deoxynojirimycin
	-O-α-d-galactopyranosyl
	-deoxynojirimycin
	Fagomine
	Rutin
	Quercetin
	Isoquercitrin
	Quercetin 3-O-glucuronide
	β-sitosterol
	Stigmasterol
	Camposterol
Saposhnikovia Root (*Saposhnikovia divaricata*)	5-O-methylvisammioside
	prim-O-glucosylcimifugin
	Cimifugin
	sec-O-glucosylhamaudol
	Hamaudol
	Lignoceric acid
	Dacursin
Mongolian Snakegourd Fruit (*Trichosanthes kirilowii*)	Bryonolic acid
	Cucurbitacin B
	Cucurbitacin D
	23, 24-Dihydrocucurbitacin B
Indigowoad Root (*Isatis indigotica*)	Epigoitrin
	Indigotin
	Indirubin
	β-sitosterol
	Clionasterol
	Sinigrin
	Indoxyl β-d-glucoside
	Epigoitrin
	Palmitic acid
	Adenosine
Baked Liquorice Root (*Glycyrrhiza glabra*)	Glycyrrhetic acid
	Glycyrrhizic acid
	Glabrolide
	Liquiritin
	Liquiritingenin
	Isoliquiritin
	Isoliquiritingenin
Magnolia Bark (*Magnolia officinalis*)	Magnolol
	Honokiol
	α-Eudesmol
	β-Eudesmol
Peppermint Herb (*Mentha haplocalyx*)	Menthol
	Menthone
	Glucoside
Fineleaf Nepeta (*Nepeta tenuifolia*)	Apigenin
	Rutin
	Kaempferol
	Chlorogenic acid
	β-Sitosterol
	Caryophyllene
	Pulegone
	Menthone
	a-Phytosterol
	α-Tocopherolquinone
