# Supplementary figures and images for: In Silico and In Vitro studies of taiwan chingguan yihau (NRICM101) on TNF-α/IL-1β-induced human lung cells
Source: Biomedicine (Taipei). 2022 Sep 1;12(3):56–71. doi: 10.37796/2211-8039.1378 (PMC9629402; doi:10.37796/2211-8039.1378)

## Slide 1
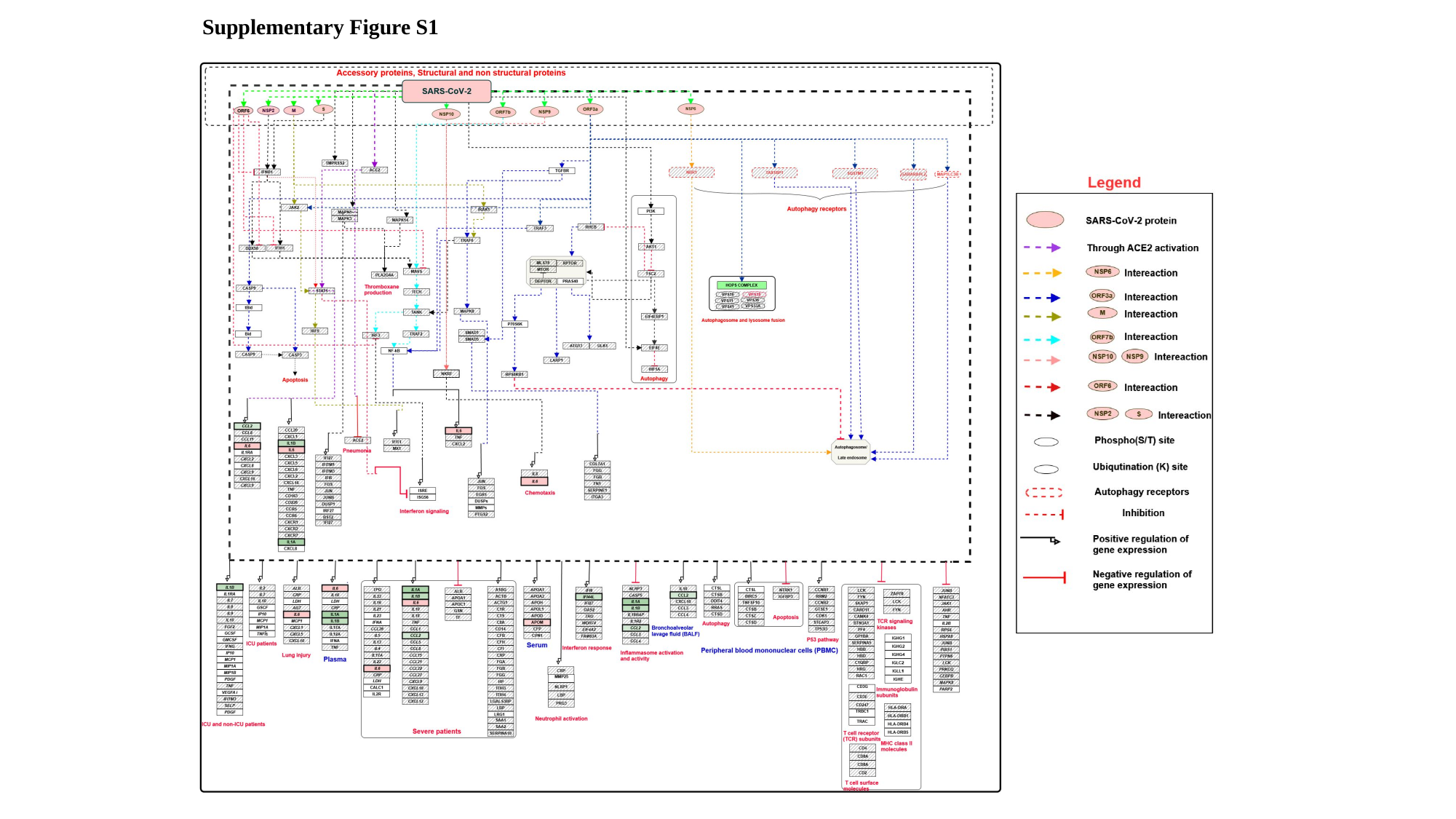

Supplementary Figure S1

Supplement: Supplementary file 1 [file BMED-12-03-056-s001.pptx]
